# The value of teachable moments in surgical patient care and the supportive role of digital technologies

**DOI:** 10.1186/s13741-019-0133-z

**Published:** 2020-02-04

**Authors:** Anna Robinson, Robert Slight, Andrew Husband, Sarah Slight

**Affiliations:** 1 School of Pharmacy, Newcastle University, King George VI Building, Newcastle upon Tyne, NE1 7RU UK; 2 Institute of Health and Society, Newcastle University, Baddiley Clark Building, Newcastle upon Tyne, UK; 3 The Newcastle upon Tyne Hospitals NHS Foundation Trust, Queen Victoria Road, Newcastle upon Tyne, NE1 4LP UK

**Keywords:** Teachable moments, Surgery, Surgical improvement, Digital technology, Behavior change, Perioperative medicine

## Abstract

Evidence strongly supports improved outcomes following surgery when patients are more physically active, have better dietary intake, or are generally fitter prior to surgery. Having an operation is a major life event for patients, and many are not educated around what they can do as individuals to aid a speedier and more successful recovery following their operation. What if there was a time point before surgery where clinicians could inspire patients to adjust their lifestyles for the better, in order to see fewer complications after surgery? This is where the concept of teachable moments comes into play.

This commentary explores the concept of teachable moments and their value in surgical patient care and discusses the potentially under-utilized opportunities on hand to the surgical multidisciplinary team to remotely support patients using digital health technologies.

## Background

For many individuals, the decision to undergo a surgery can be as life-changing as the procedure itself. The decision involves weighing up the risks and benefits of the procedure and should not be made lightly by the clinician or patient. The decision-making alone can cause individuals to reflect on poor health behaviors that they may engage in, especially if they are contributing factors for surgery. In doing so, individuals may be motivated to change their lifestyles for the better—which is where the concept of *teachable moments* comes into play.

A teachable moment is defined as an event that creates opportunity for positive behavioral change (McBride et al., 2003; Bluethmann et al., 2015). In relation to healthcare, teachable moments are points in time where clinicians can opportunistically exploit patient insight regarding suboptimal lifestyle behaviors when they are most receptive to change (Warner, 2009). In this way, healthy behaviors can be encouraged and unhealthy behaviors discouraged in a bid to improve the likelihood of more favorable surgical outcomes.

Depending on the strength of motivating factors, behavioral change may only be transient. In the context of surgery, patients may only change behaviors in the immediate period before and/or after their procedure, for example increasing physical activity levels as part of post-surgical rehabilitation (Mayer et al., 2018; Russell et al., 2011). Yet, the teachable moment has equal potential to induce improvements on a longer-term basis too: for instance, maintaining increased physical activity levels following rehabilitation in a bid to improve overall health and reduce future disease progression or recurrence (Coleman et al., 2017). It may safely be said that, regardless of the time the behavior change is sustained for, the teachable moment holds an element of “impact” to it. It is this impact that is sufficient to trigger an individual response, leading to behavioral change.

Interestingly, this impact may differ from patient to patient, and indeed from surgery to surgery. Two individuals can be diagnosed with the same condition, have the same prognosis, and require the same surgical procedure, yet the impact of the teachable moment and length of time the behavior change is sustained for can vary widely (Kelly & Barker, 2016). Beyond the impact that the teachable moment brings, there must be an underlying element of “readiness” to commit on behalf of the individual to this behavior change.

Theories of behavior change are well researched in literature, with Prochaska’s Transtheoretical Model (also known as the Stages of Change model) being one of the most popular (Prochaska & Velicer, 1997). Although the decision to undergo surgery presents a unique opportunity to instill positive health behavior changes, not every patient is ready to change immediately. Linking to this, the transtheoretical model (TTM) proposes that each individual falls within a stage for “readiness to change” and therefore can be classified as follows: pre-contemplation (they have no interest in changing behaviors), contemplation (they are considering change), preparation (they are planning to change), action (they have adopted the new, changed behavior), and maintenance (ongoing positive health behavior, without relapse). When presented with the decision to undergo surgery, it is often unclear which stage the individual may fit into this cycle of change. The value of exploiting the teachable moment rings true here; it highlights the benefits that may arise if clinicians make good use of the teachable moment to empower and educate patients. Encouraging patients to assess their current lifestyle behaviors, and explaining the underlying benefits that may arise from changing them, may trigger an individual to take a positive step towards “action.”

What is certain about behavior change and the theories underpinning *why* individuals change their health behaviors is that it is difficult to sustain a change to what can be a “lifetime habit.” If exploited correctly, the surgical teachable moment can trigger the dissolution of these habits. For instance, it is well-evidenced that smoking is associated with poorer surgical outcomes and is directly linked to increased complications, such as reduced wound healing and increased mortality (McBride et al., 2003; Webb et al., 2013). Statistics have shown that, per annum, only 3–5% of smokers in the USA spontaneously quit smoking of their own accord (Webb et al., 2013; Shi & Warner, 2010). However, in smokers who required surgery, there have been reported post-operative success rates of up to 50% following advice from surgical clinicians (Webb et al., 2013). These statistics indicate an underlying teachable moment or “trigger” proximal to the surgical decision which spurs individuals to evaluate their health behaviors and habits, motivating a change. This demonstrates how powerful capitalizing on the teachable moment can be; any intervention that exploits this can influence and motivate a significant proportion of patients.

Historically, clinicians involved in the provision of surgical care have not taken advantage of the value that surgical teachable moments offer. Time pressures and personal experience are well-known barriers to the provision of health behavior counseling (McBride et al., 2003; Grocott et al., 2017; Williams et al., 2015). The Royal College of Anaesthetists produced a report in 2019 that showcased work being done across England to improve patient care before, during, and after surgery (Royal College Of Anaesthetists, 2019). The College recognized the receptiveness of patients within their surgical journey and the benefits to individual health, public health, and socioeconomic return that could arise from improved lifestyles and physical well-being. The report acknowledges the forward-thinking vision of capitalizing on surgical teachable moments to support positive health behavior change in patients. Considering the range of healthcare professionals in the surgical multidisciplinary team, there are multiple opportunities to utilize teachable moments to provide behavior change advice to patients at various time-points along their surgical journey. However, this is not readily done (Grocott et al., 2017).

One way to *carpe diem* with respect to teachable moments may be through the use of digital technologies. Smartphone applications (apps), text messages, wearable activity trackers, and Internet-based e-platforms are all examples of interventions that have been delivered to elective patients within their surgical pathway. Combinations of text messaging and apps have delivered weight-based educational modules to pre-operative bariatric patients (Mundi et al., 2015), a web-based platform has provided dietary advice to post-operative breast cancer patients (Lee et al., 2014), and videoconferencing has supported physical rehabilitation post-orthopedic surgery (Russell et al., 2011). Studies have acknowledged patient receptiveness towards using digital technologies to complement care, resulting in behavioral change, improved recovery time, and reduced length of stay in the hospital (Grocott et al., 2017; Wynter-Blyth & Moorthy, 2017; Wolfstadt et al., 2019). Digital technologies have empowered patients to play active roles in their surgical recoveries (Lee et al., 2014); however, fewer studies have implemented technologies to support patients pre-operatively. Evidence demonstrates that digital technologies can improve a patient’s physical and psychological preparedness prior to surgery (Lemanu et al., 2018). With this in mind, perhaps technologies should be implemented earlier in the surgical pathway, to best support patients in exploiting the teachable moment.

## Conclusions

If utilized correctly, the teachable moment appears to be a powerful concept to promote health and well-being amongst a surgical population. Specifically, the surgical teachable moment presents a salient window of opportunity to address poor health behaviors that closely impact outcomes. Healthcare professionals should harness the impact of a teachable moment and capitalize on patient receptiveness to drive the improvement and quality of surgical care. Realistically, there may be limitations concerning time and staffing that may make it difficult to address health behaviors as part of inpatient elective surgical care. However, the opportunity to remotely support individuals with digital technologies and capitalize on the teachable moment pre- and post-surgery should not be ignored. Members of the perioperative team should acknowledge the value of teachable moments and recognize the supportive role of digital technologies in optimizing patient outcomes and driving healthcare delivery in a modern world.

## Data Availability

Not applicable.

## Abbreviations

TTM: Transtheoretical model of behavioral change
